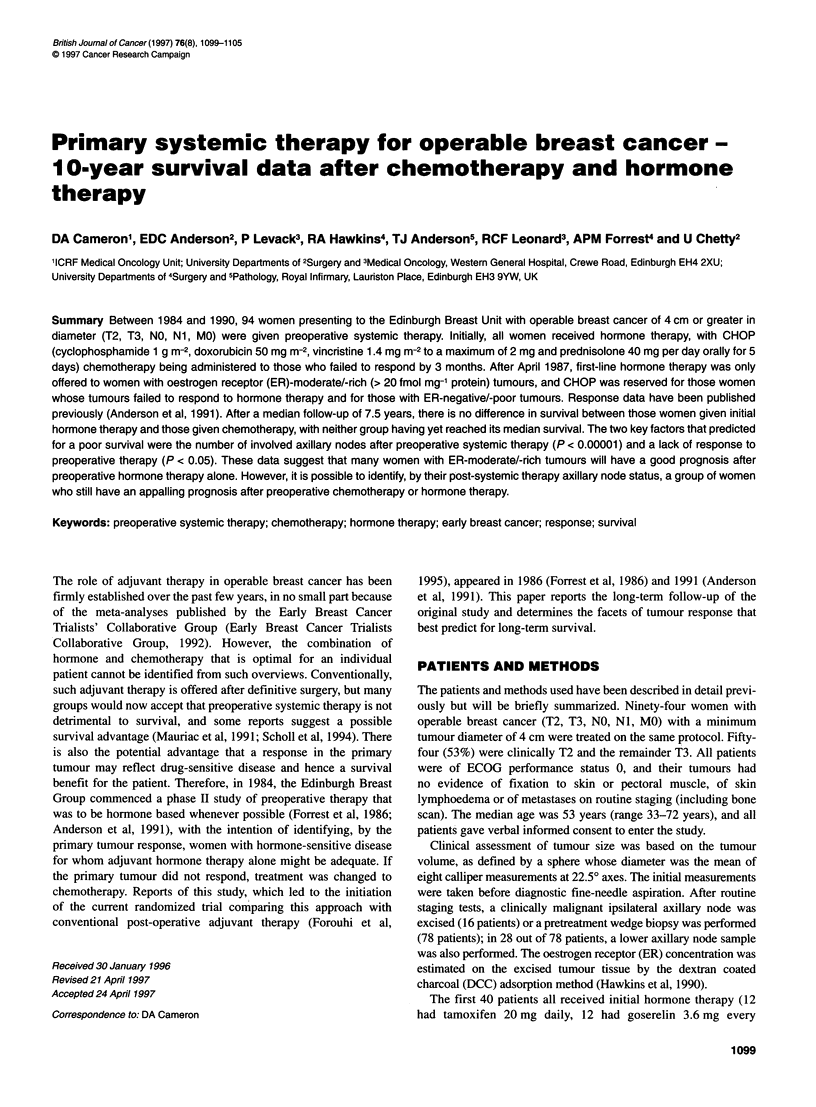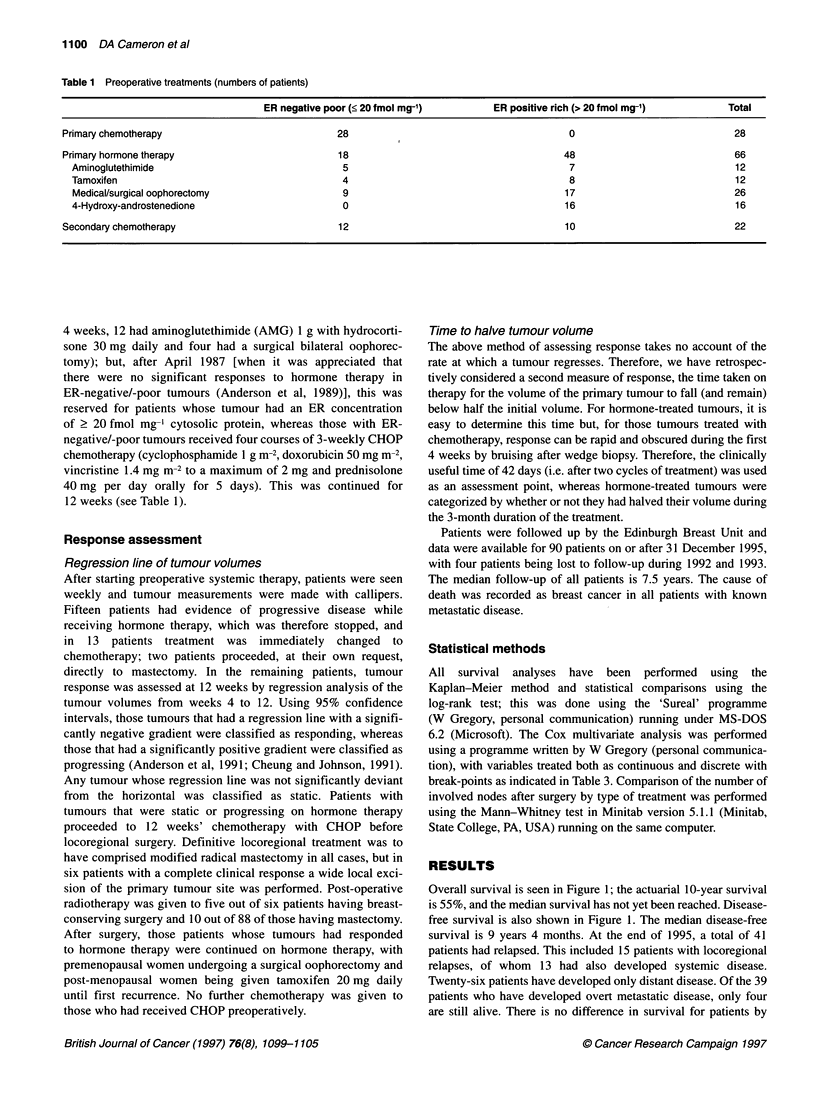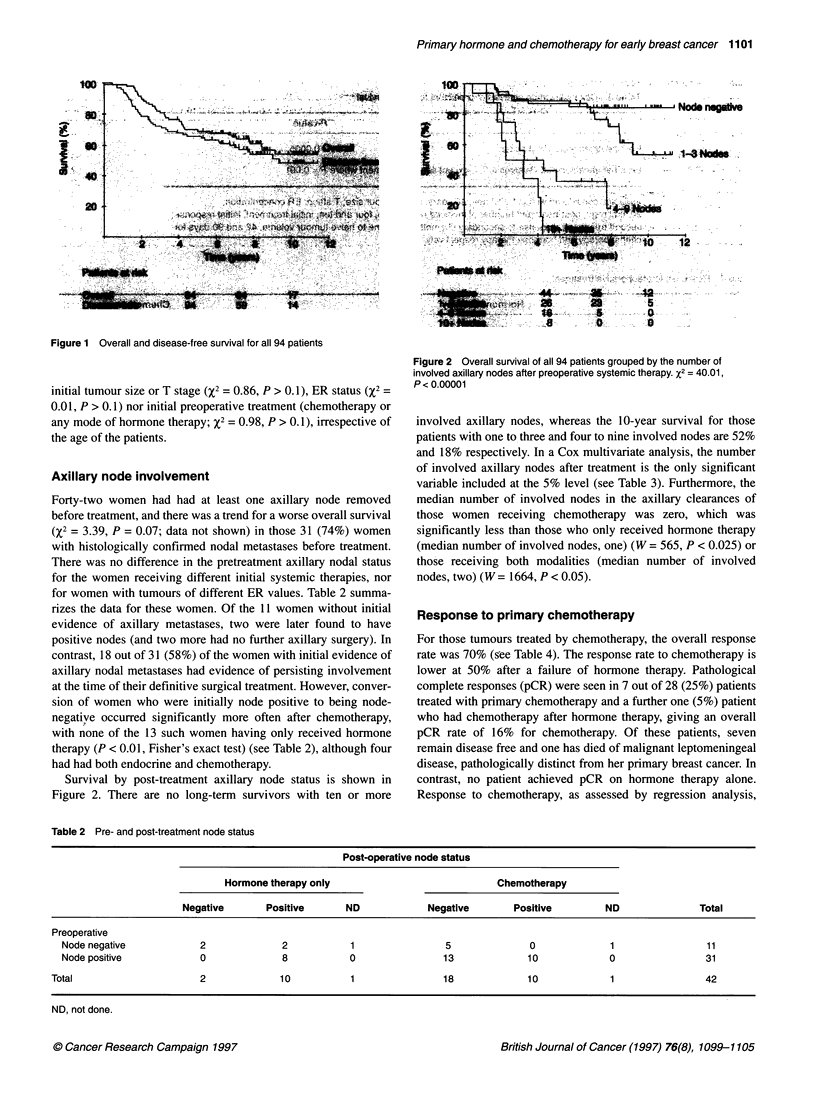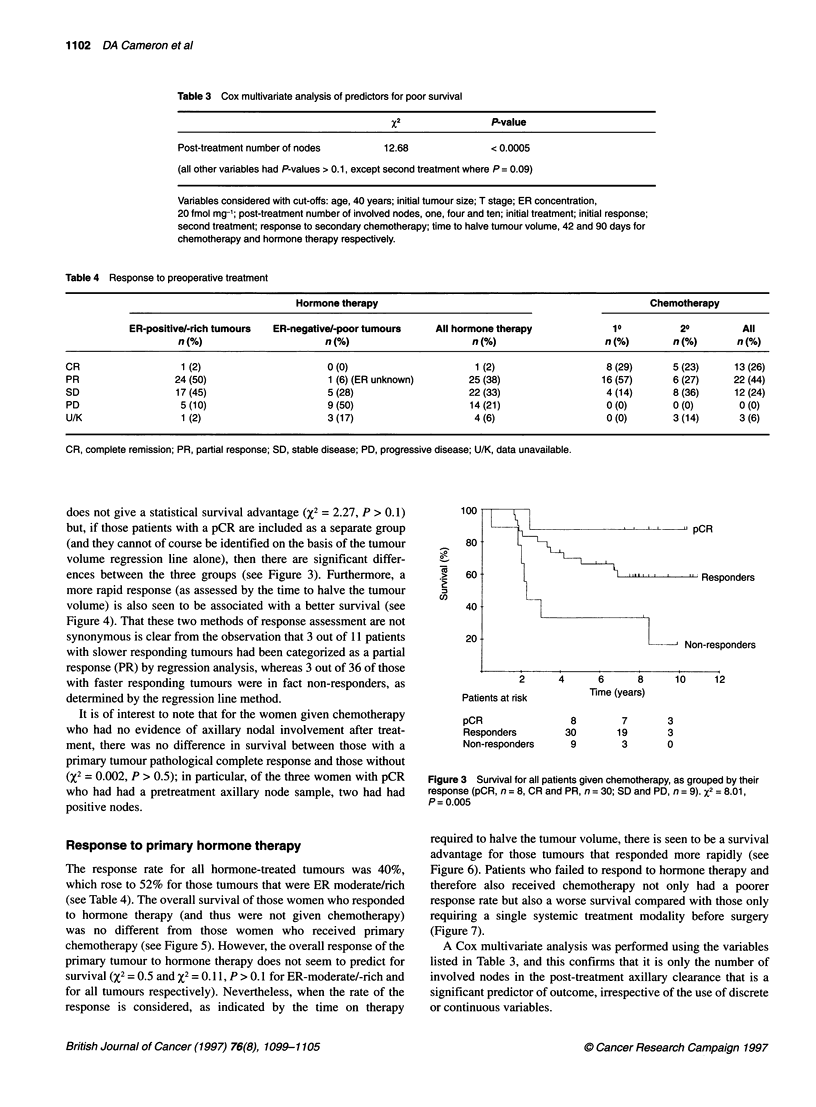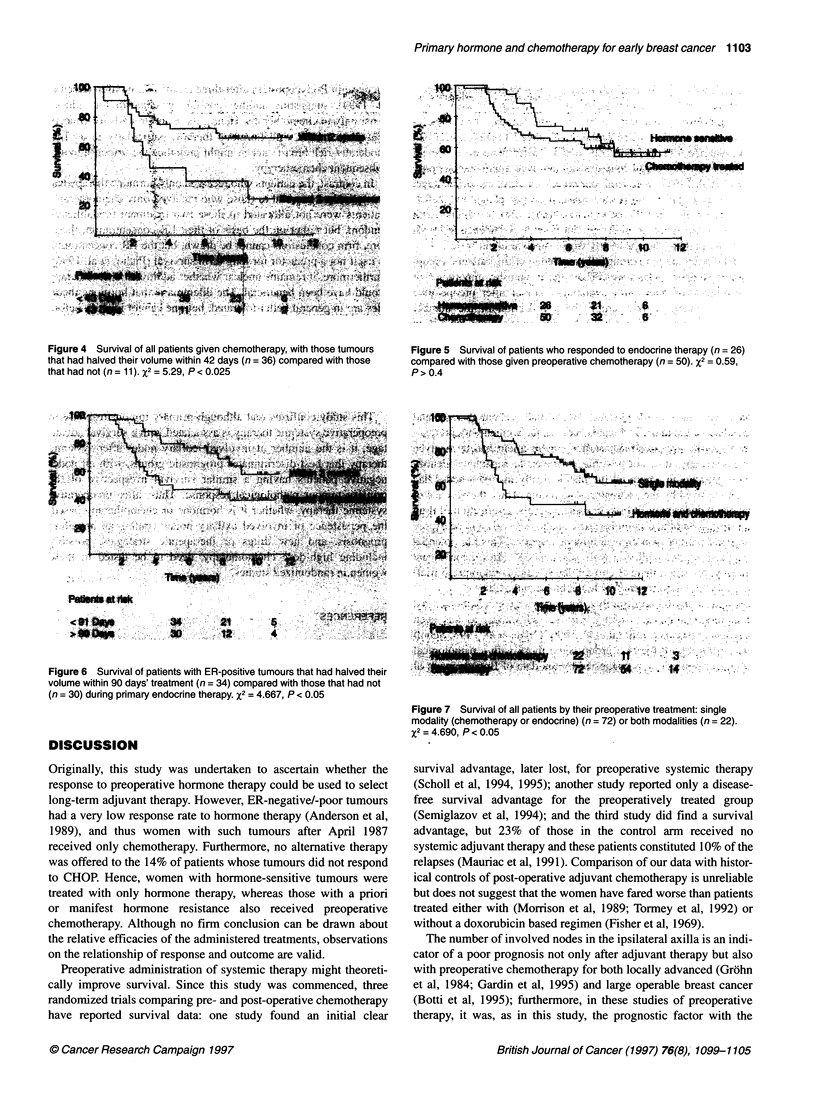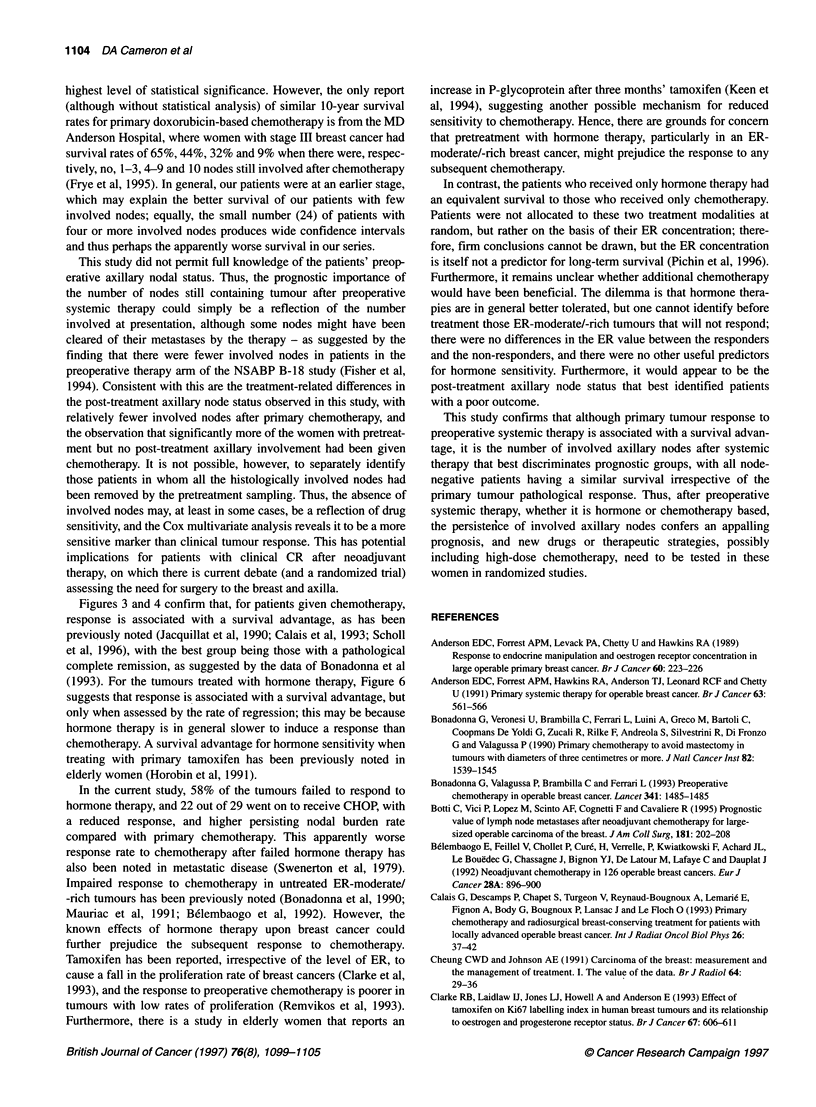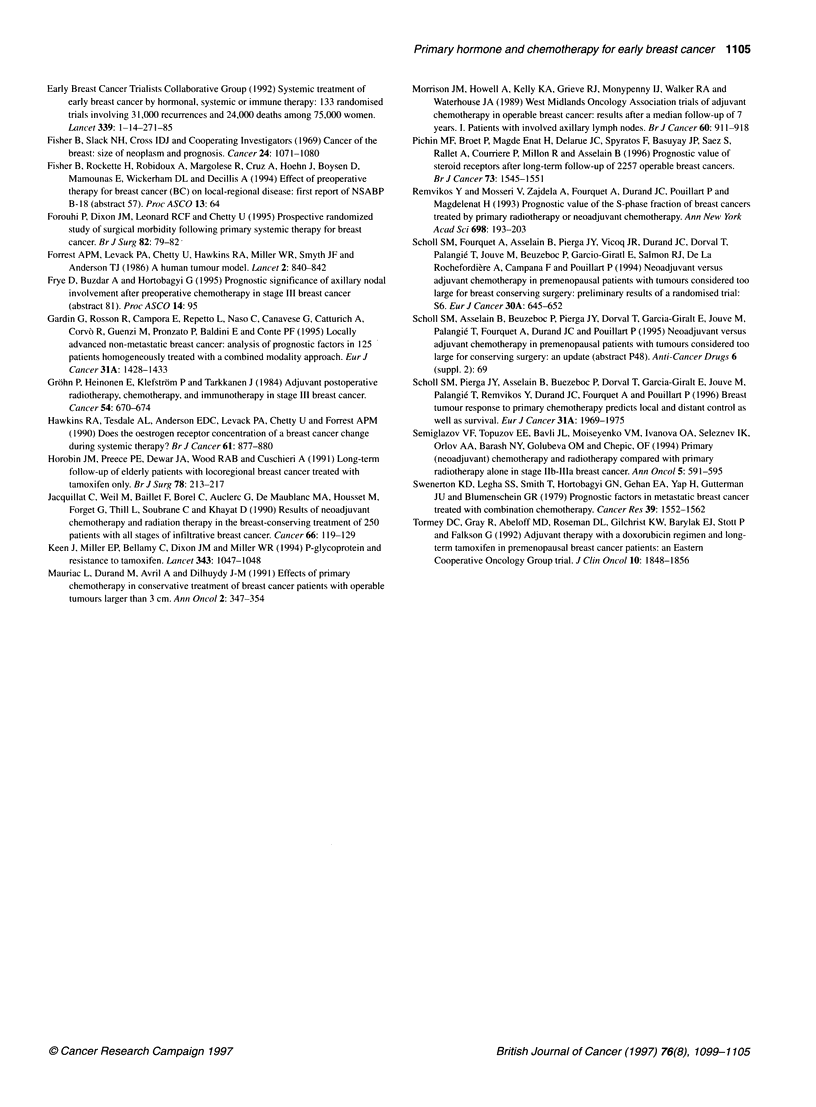# Primary systemic therapy for operable breast cancer--10-year survival data after chemotherapy and hormone therapy.

**DOI:** 10.1038/bjc.1997.514

**Published:** 1997

**Authors:** D. A. Cameron, E. D. Anderson, P. Levack, R. A. Hawkins, T. J. Anderson, R. C. Leonard, A. P. Forrest, U. Chetty

**Affiliations:** ICRF Medical Oncology Unit, Western General Hospital, Edinburgh, UK.

## Abstract

Between 1984 and 1990, 94 women presenting to the Edinburgh Breast Unit with operable breast cancer of 4 cm or greater in diameter (T2, T3, N0, N1, M0) were given preoperative systemic therapy. Initially, all women received hormone therapy, with CHOP (cyclophosphamide 1 g m(-2), doxorubicin 50 mg m(-2), vincristine 1.4 mg m(-2) to a maximum of 2 mg and prednisolone 40 mg per day orally for 5 days) chemotherapy being administered to those who failed to respond by 3 months. After April 1987, first-line hormone therapy was only offered to women with oestrogen receptor (ER)-moderate/-rich (> 20 fmol mg(-1) protein) tumours, and CHOP was reserved for those women whose tumours failed to respond to hormone therapy and for those with ER-negative/-poor tumours. Response data have been published previously (Anderson et al, 1991). After a median follow-up of 7.5 years, there is no difference in survival between those women given initial hormone therapy and those given chemotherapy, with neither group having yet reached its median survival. The two key factors that predicted for a poor survival were the number of involved axillary nodes after preoperative systemic therapy (P < 0.00001) and a lack of response to preoperative therapy (P < 0.05). These data suggest that many women with ER-moderate/-rich tumours will have a good prognosis after preoperative hormone therapy alone. However, it is possible to identify, by their post-systemic therapy axillary node status, a group of women who still have an appalling prognosis after preoperative chemotherapy or hormone therapy.


British Journal of Cancer (1997) 76(8), 1099–1105
© 1997 Cancer Research Campaign

### Primary systemic therapy for operable breast cancer – 10-year survival data after chemotherapy and hormone therapy

**DA Cameron[1], EDC Anderson[2], P Levack[3], RA Hawkins[4], TJ Anderson[5], RCF Leonard[3], APM Forrest[4] and U Chetty[2]**

[1]ICRF Medical Oncology Unit; University Departments of [2]Surgery and [3]Medical Oncology, Western General Hospital, Crewe Road, Edinburgh EH4 2XU; University Departments of [4]Surgery and [5]Pathology, Royal Infirmary, Lauriston Place, Edinburgh EH3 9YW, UK

**Summary** Between 1984 and 1990, 94 women presenting to the Edinburgh Breast Unit with operable breast cancer of 4 cm or greater in diameter (T2, T3, N0, N1, M0) were given preoperative systemic therapy. Initially, all women received hormone therapy, with CHOP (cyclophosphamide 1 g $m^{-2}$, doxorubicin 50 mg $m^{-2}$, vincristine 1.4 mg $m^{-2}$ to a maximum of 2 mg and prednisolone 40 mg per day orally for 5 days) chemotherapy being administered to those who failed to respond by 3 months. After April 1987, first-line hormone therapy was only offered to women with oestrogen receptor (ER)-moderate/-rich (> 20 fmol $mg^{-1}$ protein) tumours, and CHOP was reserved for those women whose tumours failed to respond to hormone therapy and for those with ER-negative/-poor tumours. Response data have been published previously (Anderson et al, 1991). After a median follow-up of 7.5 years, there is no difference in survival between those women given initial hormone therapy and those given chemotherapy, with neither group having yet reached its median survival. The two key factors that predicted for a poor survival were the number of involved axillary nodes after preoperative systemic therapy ($P < 0.00001$) and a lack of response to preoperative therapy ($P < 0.05$). These data suggest that many women with ER-moderate/-rich tumours will have a good prognosis after preoperative hormone therapy alone. However, it is possible to identify, by their post-systemic therapy axillary node status, a group of women who still have an appalling prognosis after preoperative chemotherapy or hormone therapy.

**Keywords:** preoperative systemic therapy; chemotherapy; hormone therapy; early breast cancer; response; survival

The role of adjuvant therapy in operable breast cancer has been firmly established over the past few years, in no small part because of the meta-analyses published by the Early Breast Cancer Trialists' Collaborative Group (Early Breast Cancer Trialists Collaborative Group, 1992). However, the combination of hormone and chemotherapy that is optimal for an individual patient cannot be identified from such overviews. Conventionally, such adjuvant therapy is offered after definitive surgery, but many groups would now accept that preoperative systemic therapy is not detrimental to survival, and some reports suggest a possible survival advantage (Mauriac et al, 1991; Scholl et al, 1994). There is also the potential advantage that a response in the primary tumour may reflect drug-sensitive disease and hence a survival benefit for the patient. Therefore, in 1984, the Edinburgh Breast Group commenced a phase II study of preoperative therapy that was to be hormone based whenever possible (Forrest et al, 1986; Anderson et al, 1991), with the intention of identifying, by the primary tumour response, women with hormone-sensitive disease for whom adjuvant hormone therapy alone might be adequate. If the primary tumour did not respond, treatment was changed to chemotherapy. Reports of this study, which led to the initiation of the current randomized trial comparing this approach with conventional post-operative adjuvant therapy (Forouhi et al, 1995), appeared in 1986 (Forrest et al, 1986) and 1991 (Anderson et al, 1991). This paper reports the long-term follow-up of the original study and determines the facets of tumour response that best predict for long-term survival.

*Received 30 January 1996*
*Revised 21 April 1997*
*Accepted 24 April 1997*

*Correspondence to:* DA Cameron

### PATIENTS AND METHODS

The patients and methods used have been described in detail previously but will be briefly summarized. Ninety-four women with operable breast cancer (T2, T3, N0, N1, M0) with a minimum tumour diameter of 4 cm were treated on the same protocol. Fifty-four (53%) were clinically T2 and the remainder T3. All patients were of ECOG performance status 0, and their tumours had no evidence of fixation to skin or pectoral muscle, of skin lymphoedema or of metastases on routine staging (including bone scan). The median age was 53 years (range 33–72 years), and all patients gave verbal informed consent to enter the study.

Clinical assessment of tumour size was based on the tumour volume, as defined by a sphere whose diameter was the mean of eight calliper measurements at 22.5° axes. The initial measurements were taken before diagnostic fine-needle aspiration. After routine staging tests, a clinically malignant ipsilateral axillary node was excised (16 patients) or a pretreatment wedge biopsy was performed (78 patients); in 28 out of 78 patients, a lower axillary node sample was also performed. The oestrogen receptor (ER) concentration was estimated on the excised tumour tissue by the dextran coated charcoal (DCC) adsorption method (Hawkins et al, 1990).

The first 40 patients all received initial hormone therapy (12 had tamoxifen 20 mg daily, 12 had goserelin 3.6 mg every

**Table 1** Preoperative treatments (numbers of patients)

| | ER negative poor (≤ 20 fmol mg$^{-1}$) | ER positive rich (> 20 fmol mg$^{-1}$) | Total |
|---|---|---|---|
| Primary chemotherapy | 28 | 0 | 28 |
| Primary hormone therapy | 18 | 48 | 66 |
| Aminoglutethimide | 5 | 7 | 12 |
| Tamoxifen | 4 | 8 | 12 |
| Medical/surgical oophorectomy | 9 | 17 | 26 |
| 4-Hydroxy-androstenedione | 0 | 16 | 16 |
| Secondary chemotherapy | 12 | 10 | 22 |

4 weeks, 12 had aminoglutethimide (AMG) 1 g with hydrocortisone 30 mg daily and four had a surgical bilateral oophorectomy); but, after April 1987 [when it was appreciated that there were no significant responses to hormone therapy in ER-negative/-poor tumours (Anderson et al, 1989)], this was reserved for patients whose tumour had an ER concentration of ≥ 20 fmol mg$^{-1}$ cytosolic protein, whereas those with ER-negative/-poor tumours received four courses of 3-weekly CHOP chemotherapy (cyclophosphamide 1 g m$^{-2}$, doxorubicin 50 mg m$^{-2}$, vincristine 1.4 mg m$^{-2}$ to a maximum of 2 mg and prednisolone 40 mg per day orally for 5 days). This was continued for 12 weeks (see Table 1).

### Response assessment

### *Regression line of tumour volumes*

After starting preoperative systemic therapy, patients were seen weekly and tumour measurements were made with callipers. Fifteen patients had evidence of progressive disease while receiving hormone therapy, which was therefore stopped, and in 13 patients treatment was immediately changed to chemotherapy; two patients proceeded, at their own request, directly to mastectomy. In the remaining patients, tumour response was assessed at 12 weeks by regression analysis of the tumour volumes from weeks 4 to 12. Using 95% confidence intervals, those tumours that had a regression line with a significantly negative gradient were classified as responding, whereas those that had a significantly positive gradient were classified as progressing (Anderson et al, 1991; Cheung and Johnson, 1991). Any tumour whose regression line was not significantly deviant from the horizontal was classified as static. Patients with tumours that were static or progressing on hormone therapy proceeded to 12 weeks' chemotherapy with CHOP before locoregional surgery. Definitive locoregional treatment was to have comprised modified radical mastectomy in all cases, but in six patients with a complete clinical response a wide local excision of the primary tumour site was performed. Post-operative radiotherapy was given to five out of six patients having breast-conserving surgery and 10 out of 88 of those having mastectomy. After surgery, those patients whose tumours had responded to hormone therapy were continued on hormone therapy, with premenopausal women undergoing a surgical oophorectomy and post-menopausal women being given tamoxifen 20 mg daily until first recurrence. No further chemotherapy was given to those who had received CHOP preoperatively.

### *Time to halve tumour volume*

The above method of assessing response takes no account of the rate at which a tumour regresses. Therefore, we have retrospectively considered a second measure of response, the time taken on therapy for the volume of the primary tumour to fall (and remain) below half the initial volume. For hormone-treated tumours, it is easy to determine this time but, for those tumours treated with chemotherapy, response can be rapid and obscured during the first 4 weeks by bruising after wedge biopsy. Therefore, the clinically useful time of 42 days (i.e. after two cycles of treatment) was used as an assessment point, whereas hormone-treated tumours were categorized by whether or not they had halved their volume during the 3-month duration of the treatment.

Patients were followed up by the Edinburgh Breast Unit and data were available for 90 patients on or after 31 December 1995, with four patients being lost to follow-up during 1992 and 1993. The median follow-up of all patients is 7.5 years. The cause of death was recorded as breast cancer in all patients with known metastatic disease.

### Statistical methods

All survival analyses have been performed using the Kaplan–Meier method and statistical comparisons using the log-rank test; this was done using the 'Sureal' programme (W Gregory, personal communication) running under MS-DOS 6.2 (Microsoft). The Cox multivariate analysis was performed using a programme written by W Gregory (personal communication), with variables treated both as continuous and discrete with break-points as indicated in Table 3. Comparison of the number of involved nodes after surgery by type of treatment was performed using the Mann–Whitney test in Minitab version 5.1.1 (Minitab, State College, PA, USA) running on the same computer.

### RESULTS

Overall survival is seen in Figure 1; the actuarial 10-year survival is 55%, and the median survival has not yet been reached. Disease-free survival is also shown in Figure 1. The median disease-free survival is 9 years 4 months. At the end of 1995, a total of 41 patients had relapsed. This included 15 patients with locoregional relapses, of whom 13 had also developed systemic disease. Twenty-six patients have developed only distant disease. Of the 39 patients who have developed overt metastatic disease, only four are still alive. There is no difference in survival for patients by

 © Cancer Research Campaign 1997

**Figure 1** Overall and disease-free survival for all 94 patients

**Figure 2** Overall survival of all 94 patients grouped by the number of involved axillary nodes after preoperative systemic therapy. $\chi^2 = 40.01$, $P < 0.00001$

initial tumour size or T stage ($\chi^2 = 0.86$, $P > 0.1$), ER status ($\chi^2 = 0.01$, $P > 0.1$) nor initial preoperative treatment (chemotherapy or any mode of hormone therapy; $\chi^2 = 0.98$, $P > 0.1$), irrespective of the age of the patients.

### Axillary node involvement

Forty-two women had had at least one axillary node removed before treatment, and there was a trend for a worse overall survival ($\chi^2 = 3.39$, $P = 0.07$; data not shown) in those 31 (74%) women with histologically confirmed nodal metastases before treatment. There was no difference in the pretreatment axillary nodal status for the women receiving different initial systemic therapies, nor for women with tumours of different ER values. Table 2 summarizes the data for these women. Of the 11 women without initial evidence of axillary metastases, two were later found to have positive nodes (and two more had no further axillary surgery). In contrast, 18 out of 31 (58%) of the women with initial evidence of axillary nodal metastases had evidence of persisting involvement at the time of their definitive surgical treatment. However, conversion of women who were initially node positive to being node-negative occurred significantly more often after chemotherapy, with none of the 13 such women having only received hormone therapy ($P < 0.01$, Fisher's exact test) (see Table 2), although four had had both endocrine and chemotherapy.

Survival by post-treatment axillary node status is shown in Figure 2. There are no long-term survivors with ten or more involved axillary nodes, whereas the 10-year survival for those patients with one to three and four to nine involved nodes are 52% and 18% respectively. In a Cox multivariate analysis, the number of involved axillary nodes after treatment is the only significant variable included at the 5% level (see Table 3). Furthermore, the median number of involved nodes in the axillary clearances of those women receiving chemotherapy was zero, which was significantly less than those who only received hormone therapy (median number of involved nodes, one) ($W = 565$, $P < 0.025$) or those receiving both modalities (median number of involved nodes, two) ($W = 1664$, $P < 0.05$).

### Response to primary chemotherapy

For those tumours treated by chemotherapy, the overall response rate was 70% (see Table 4). The response rate to chemotherapy is lower at 50% after a failure of hormone therapy. Pathological complete responses (pCR) were seen in 7 out of 28 (25%) patients treated with primary chemotherapy and a further one (5%) patient who had chemotherapy after hormone therapy, giving an overall pCR rate of 16% for chemotherapy. Of these patients, seven remain disease free and one has died of malignant leptomeningeal disease, pathologically distinct from her primary breast cancer. In contrast, no patient achieved pCR on hormone therapy alone. Response to chemotherapy, as assessed by regression analysis,

**Table 2** Pre- and post-treatment node status

| | Post-operative node status | | | | | | |
|---|---|---|---|---|---|---|---|
| | Hormone therapy only | | | Chemotherapy | | | |
| | Negative | Positive | ND | Negative | Positive | ND | Total |
| Preoperative | | | | | | | |
| Node negative | 2 | 2 | 1 | 5 | 0 | 1 | 11 |
| Node positive | 0 | 8 | 0 | 13 | 10 | 0 | 31 |
| Total | 2 | 10 | 1 | 18 | 10 | 1 | 42 |

ND, not done.

**Table 3** Cox multivariate analysis of predictors for poor survival

| | $\chi^2$ | *P*-value |
|---|---|---|
| Post-treatment number of nodes | 12.68 | < 0.0005 |
| (all other variables had *P*-values > 0.1, except second treatment where *P* = 0.09) | | |

Variables considered with cut-offs: age, 40 years; initial tumour size; T stage; ER concentration, 20 fmol mg$^{-1}$; post-treatment number of involved nodes, one, four and ten; initial treatment; initial response; second treatment; response to secondary chemotherapy; time to halve tumour volume, 42 and 90 days for chemotherapy and hormone therapy respectively.

**Table 4** Response to preoperative treatment

| | Hormone therapy | | | Chemotherapy | | |
|---|---|---|---|---|---|---|
| | ER-positive/-rich tumours *n* (%) | ER-negative/-poor tumours *n* (%) | All hormone therapy *n* (%) | 1° *n* (%) | 2° *n* (%) | All *n* (%) |
| CR | 1 (2) | 0 (0) | 1 (2) | 8 (29) | 5 (23) | 13 (26) |
| PR | 24 (50) | 1 (6) (ER unknown) | 25 (38) | 16 (57) | 6 (27) | 22 (44) |
| SD | 17 (45) | 5 (28) | 22 (33) | 4 (14) | 8 (36) | 12 (24) |
| PD | 5 (10) | 9 (50) | 14 (21) | 0 (0) | 0 (0) | 0 (0) |
| U/K | 1 (2) | 3 (17) | 4 (6) | 0 (0) | 3 (14) | 3 (6) |

CR, complete remission; PR, partial response; SD, stable disease; PD, progressive disease; U/K, data unavailable.

does not give a statistical survival advantage ($\chi^2 = 2.27$, $P > 0.1$) but, if those patients with a pCR are included as a separate group (and they cannot of course be identified on the basis of the tumour volume regression line alone), then there are significant differences between the three groups (see Figure 3). Furthermore, a more rapid response (as assessed by the time to halve the tumour volume) is also seen to be associated with a better survival (see Figure 4). That these two methods of response assessment are not synonymous is clear from the observation that 3 out of 11 patients with slower responding tumours had been categorized as a partial response (PR) by regression analysis, whereas 3 out of 36 of those with faster responding tumours were in fact non-responders, as determined by the regression line method.

It is of interest to note that for the women given chemotherapy who had no evidence of axillary nodal involvement after treatment, there was no difference in survival between those with a primary tumour pathological complete response and those without ($\chi^2 = 0.002$, $P > 0.5$); in particular, of the three women with pCR who had had a pretreatment axillary node sample, two had had positive nodes.

**Figure 3** Survival for all patients given chemotherapy, as grouped by their response (pCR, *n* = 8, CR and PR, *n* = 30; SD and PD, *n* = 9). $\chi^2 = 8.01$, $P = 0.005$

### Response to primary hormone therapy

The response rate for all hormone-treated tumours was 40%, which rose to 52% for those tumours that were ER moderate/rich (see Table 4). The overall survival of those women who responded to hormone therapy (and thus were not given chemotherapy) was no different from those women who received primary chemotherapy (see Figure 5). However, the overall response of the primary tumour to hormone therapy does not seem to predict for survival ($\chi^2 = 0.5$ and $\chi^2 = 0.11$, $P > 0.1$ for ER-moderate/-rich and for all tumours respectively). Nevertheless, when the rate of the response is considered, as indicated by the time on therapy required to halve the tumour volume, there is seen to be a survival advantage for those tumours that responded more rapidly (see Figure 6). Patients who failed to respond to hormone therapy and therefore also received chemotherapy not only had a poorer response rate but also a worse survival compared with those only requiring a single systemic treatment modality before surgery (Figure 7).

A Cox multivariate analysis was performed using the variables listed in Table 3, and this confirms that it is only the number of involved nodes in the post-treatment axillary clearance that is a significant predictor of outcome, irrespective of the use of discrete or continuous variables.

 © Cancer Research Campaign 1997

**Figure 4** Survival of all patients given chemotherapy, with those tumours that had halved their volume within 42 days ($n = 36$) compared with those that had not ($n = 11$). $\chi^2 = 5.29$, $P < 0.025$

**Figure 5** Survival of patients who responded to endocrine therapy ($n = 26$) compared with those given preoperative chemotherapy ($n = 50$). $\chi^2 = 0.59$, $P > 0.4$

**Figure 6** Survival of patients with ER-positive tumours that had halved their volume within 90 days' treatment ($n = 34$) compared with those that had not ($n = 30$) during primary endocrine therapy. $\chi^2 = 4.667$, $P < 0.05$

**Figure 7** Survival of all patients by their preoperative treatment: single modality (chemotherapy or endocrine) ($n = 72$) or both modalities ($n = 22$). $\chi^2 = 4.690$, $P < 0.05$

### DISCUSSION

Originally, this study was undertaken to ascertain whether the response to preoperative hormone therapy could be used to select long-term adjuvant therapy. However, ER-negative/-poor tumours had a very low response rate to hormone therapy (Anderson et al, 1989), and thus women with such tumours after April 1987 received only chemotherapy. Furthermore, no alternative therapy was offered to the 14% of patients whose tumours did not respond to CHOP. Hence, women with hormone-sensitive tumours were treated with only hormone therapy, whereas those with a priori or manifest hormone resistance also received preoperative chemotherapy. Although no firm conclusion can be drawn about the relative efficacies of the administered treatments, observations on the relationship of response and outcome are valid.

Preoperative administration of systemic therapy might theoretically improve survival. Since this study was commenced, three randomized trials comparing pre- and post-operative chemotherapy have reported survival data: one study found an initial clear survival advantage, later lost, for preoperative systemic therapy (Scholl et al, 1994, 1995); another study reported only a disease-free survival advantage for the preoperatively treated group (Semiglazov et al, 1994); and the third study did find a survival advantage, but 23% of those in the control arm received no systemic adjuvant therapy and these patients constituted 10% of the relapses (Mauriac et al, 1991). Comparison of our data with historical controls of post-operative adjuvant chemotherapy is unreliable but does not suggest that the women have fared worse than patients treated either with (Morrison et al, 1989; Tormey et al, 1992) or without a doxorubicin based regimen (Fisher et al, 1969).

The number of involved nodes in the ipsilateral axilla is an indicator of a poor prognosis not only after adjuvant therapy but also with preoperative chemotherapy for both locally advanced (Gröhn et al, 1984; Gardin et al, 1995) and large operable breast cancer (Botti et al, 1995); furthermore, in these studies of preoperative therapy, it was, as in this study, the prognostic factor with the

© Cancer Research Campaign 1997

highest level of statistical significance. However, the only report (although without statistical analysis) of similar 10-year survival rates for primary doxorubicin-based chemotherapy is from the MD Anderson Hospital, where women with stage III breast cancer had survival rates of 65%, 44%, 32% and 9% when there were, respectively, no, 1–3, 4–9 and 10 nodes still involved after chemotherapy (Frye et al, 1995). In general, our patients were at an earlier stage, which may explain the better survival of our patients with few involved nodes; equally, the small number (24) of patients with four or more involved nodes produces wide confidence intervals and thus perhaps the apparently worse survival in our series.

This study did not permit full knowledge of the patients' preoperative axillary nodal status. Thus, the prognostic importance of the number of nodes still containing tumour after preoperative systemic therapy could simply be a reflection of the number involved at presentation, although some nodes might have been cleared of their metastases by the therapy – as suggested by the finding that there were fewer involved nodes in patients in the preoperative therapy arm of the NSABP B-18 study (Fisher et al, 1994). Consistent with this are the treatment-related differences in the post-treatment axillary node status observed in this study, with relatively fewer involved nodes after primary chemotherapy, and the observation that significantly more of the women with pretreatment but no post-treatment axillary involvement had been given chemotherapy. It is not possible, however, to separately identify those patients in whom all the histologically involved nodes had been removed by the pretreatment sampling. Thus, the absence of involved nodes may, at least in some cases, be a reflection of drug sensitivity, and the Cox multivariate analysis reveals it to be a more sensitive marker than clinical tumour response. This has potential implications for patients with clinical CR after neoadjuvant therapy, on which there is current debate (and a randomized trial) assessing the need for surgery to the breast and axilla.

Figures 3 and 4 confirm that, for patients given chemotherapy, response is associated with a survival advantage, as has been previously noted (Jacquillat et al, 1990; Calais et al, 1993; Scholl et al, 1996), with the best group being those with a pathological complete remission, as suggested by the data of Bonadonna et al (1993). For the tumours treated with hormone therapy, Figure 6 suggests that response is associated with a survival advantage, but only when assessed by the rate of regression; this may be because hormone therapy is in general slower to induce a response than chemotherapy. A survival advantage for hormone sensitivity when treating with primary tamoxifen has been previously noted in elderly women (Horobin et al, 1991).

In the current study, 58% of the tumours failed to respond to hormone therapy, and 22 out of 29 went on to receive CHOP, with a reduced response, and higher persisting nodal burden rate compared with primary chemotherapy. This apparently worse response rate to chemotherapy after failed hormone therapy has also been noted in metastatic disease (Swenerton et al, 1979). Impaired response to chemotherapy in untreated ER-moderate/-rich tumours has been previously noted (Bonadonna et al, 1990; Mauriac et al, 1991; Bélembaogo et al, 1992). However, the known effects of hormone therapy upon breast cancer could further prejudice the subsequent response to chemotherapy. Tamoxifen has been reported, irrespective of the level of ER, to cause a fall in the proliferation rate of breast cancers (Clarke et al, 1993), and the response to preoperative chemotherapy is poorer in tumours with low rates of proliferation (Remvikos et al, 1993). Furthermore, there is a study in elderly women that reports an increase in P-glycoprotein after three months' tamoxifen (Keen et al, 1994), suggesting another possible mechanism for reduced sensitivity to chemotherapy. Hence, there are grounds for concern that pretreatment with hormone therapy, particularly in an ER-moderate/-rich breast cancer, might prejudice the response to any subsequent chemotherapy.

In contrast, the patients who received only hormone therapy had an equivalent survival to those who received only chemotherapy. Patients were not allocated to these two treatment modalities at random, but rather on the basis of their ER concentration; therefore, firm conclusions cannot be drawn, but the ER concentration is itself not a predictor for long-term survival (Pichin et al, 1996). Furthermore, it remains unclear whether additional chemotherapy would have been beneficial. The dilemma is that hormone therapies are in general better tolerated, but one cannot identify before treatment those ER-moderate/-rich tumours that will not respond; there were no differences in the ER value between the responders and the non-responders, and there were no other useful predictors for hormone sensitivity. Furthermore, it would appear to be the post-treatment axillary node status that best identified patients with a poor outcome.

This study confirms that although primary tumour response to preoperative systemic therapy is associated with a survival advantage, it is the number of involved axillary nodes after systemic therapy that best discriminates prognostic groups, with all node-negative patients having a similar survival irrespective of the primary tumour pathological response. Thus, after preoperative systemic therapy, whether it is hormone or chemotherapy based, the persistence of involved axillary nodes confers an appalling prognosis, and new drugs or therapeutic strategies, possibly including high-dose chemotherapy, need to be tested in these women in randomized studies.

### REFERENCES

Anderson EDC, Forrest APM, Levack PA, Chetty U and Hawkins RA (1989) Response to endocrine manipulation and oestrogen receptor concentration in large operable primary breast cancer. *Br J Cancer* **60**: 223–226

Anderson EDC, Forrest APM, Hawkins RA, Anderson TJ, Leonard RCF and Chetty U (1991) Primary systemic therapy for operable breast cancer. *Br J Cancer* **63**: 561–566

Bonadonna G, Veronesi U, Brambilla C, Ferrari L, Luini A, Greco M, Bartoli C, Coopmans De Yoldi G, Zucali R, Rilke F, Andreola S, Silvestrini R, Di Fronzo G and Valagussa P (1990) Primary chemotherapy to avoid mastectomy in tumours with diameters of three centimetres or more. *J Natl Cancer Inst* **82**: 1539–1545

Bonadonna G, Valagussa P, Brambilla C and Ferrari L (1993) Preoperative chemotherapy in operable breast cancer. *Lancet* **341**: 1485–1485

Botti C, Vici P, Lopez M, Scinto AF, Cognetti F and Cavaliere R (1995) Prognostic value of lymph node metastases after neoadjuvant chemotherapy for large-sized operable carcinoma of the breast. *J Am Coll Surg*, **181**: 202–208

Bélembaogo E, Feillel V, Chollet P, Curé, H, Verrelle, P, Kwiatkowski F, Achard JL, Le Bouëdec G, Chassagne J, Bignon YJ, De Latour M, Lafaye C and Dauplat J (1992) Neoadjuvant chemotherapy in 126 operable breast cancers. *Eur J Cancer* **28A**: 896–900

Calais G, Descamps P, Chapet S, Turgeon V, Reynaud-Bougnoux A, Lemarié E, Fignon A, Body G, Bougnoux P, Lansac J and Le Floch O (1993) Primary chemotherapy and radiosurgical breast-conserving treatment for patients with locally advanced operable breast cancer. *Int J Radiat Oncol Biol Phys* **26**: 37–42

Cheung CWD and Johnson AE (1991) Carcinoma of the breast: measurement and the management of treatment. I. The value of the data. *Br J Radiol* **64**: 29–36

Clarke RB, Laidlaw IJ, Jones LJ, Howell A and Anderson E (1993) Effect of tamoxifen on Ki67 labelling index in human breast tumours and its relationship to oestrogen and progesterone receptor status. *Br J Cancer* **67**: 606–611

© Cancer Research Campaign 1997

Early Breast Cancer Trialists Collaborative Group (1992) Systemic treatment of early breast cancer by hormonal, systemic or immune therapy: 133 randomised trials involving 31,000 recurrences and 24,000 deaths among 75,000 women. *Lancet* **339**: 1-14-271-85

Fisher B, Slack NH, Cross IDJ and Cooperating Investigators (1969) Cancer of the breast: size of neoplasm and prognosis. *Cancer* **24**: 1071–1080

Fisher B, Rockette H, Robidoux A, Margolese R, Cruz A, Hoehn J, Boysen D, Mamounas E, Wickerham DL and Decillis A (1994) Effect of preoperative therapy for breast cancer (BC) on local-regional disease: first report of NSABP B-18 (abstract 57). *Proc ASCO* **13**: 64

Forouhi P, Dixon JM, Leonard RCF and Chetty U (1995) Prospective randomized study of surgical morbidity following primary systemic therapy for breast cancer. *Br J Surg* **82**: 79–82

Forrest APM, Levack PA, Chetty U, Hawkins RA, Miller WR, Smyth JF and Anderson TJ (1986) A human tumour model. *Lancet* **2**: 840–842

Frye D, Buzdar A and Hortobagyi G (1995) Prognostic significance of axillary nodal involvement after preoperative chemotherapy in stage III breast cancer (abstract 81). *Proc ASCO* **14**: 95

Gardin G, Rosson R, Campora E, Repetto L, Naso C, Canavese G, Catturich A, Corvò R, Guenzi M, Pronzato P, Baldini E and Conte PF (1995) Locally advanced non-metastatic breast cancer: analysis of prognostic factors in 125 patients homogeneously treated with a combined modality approach. *Eur J Cancer* **31A**: 1428–1433

Gröhn P, Heinonen E, Klefström P and Tarkkanen J (1984) Adjuvant postoperative radiotherapy, chemotherapy, and immunotherapy in stage III breast cancer. *Cancer* **54**: 670–674

Hawkins RA, Tesdale AL, Anderson EDC, Levack PA, Chetty U and Forrest APM (1990) Does the oestrogen receptor concentration of a breast cancer change during systemic therapy? *Br J Cancer* **61**: 877–880

Horobin JM, Preece PE, Dewar JA, Wood RAB and Cuschieri A (1991) Long-term follow-up of elderly patients with locoregional breast cancer treated with tamoxifen only. *Br J Surg* **78**: 213–217

Jacquillat C, Weil M, Baillet F, Borel C, Auclerc G, De Maublanc MA, Housset M, Forget G, Thill L, Soubrane C and Khayat D (1990) Results of neoadjuvant chemotherapy and radiation therapy in the breast-conserving treatment of 250 patients with all stages of infiltrative breast cancer. *Cancer* **66**: 119–129

Keen J, Miller EP, Bellamy C, Dixon JM and Miller WR (1994) P-glycoprotein and resistance to tamoxifen. *Lancet* **343**: 1047–1048

Mauriac L, Durand M, Avril A and Dilhuydy J-M (1991) Effects of primary chemotherapy in conservative treatment of breast cancer patients with operable tumours larger than 3 cm. *Ann Oncol* **2**: 347–354

Morrison JM, Howell A, Kelly KA, Grieve RJ, Monypenny IJ, Walker RA and Waterhouse JA (1989) West Midlands Oncology Association trials of adjuvant chemotherapy in operable breast cancer: results after a median follow-up of 7 years. I. Patients with involved axillary lymph nodes. *Br J Cancer* **60**: 911–918

Pichin MF, Broet P, Magde Enat H, Delarue JC, Spyratos F, Basuyay JP, Saez S, Rallet A, Courriere P, Millon R and Asselain B (1996) Prognostic value of steroid receptors after long-term follow-up of 2257 operable breast cancers. *Br J Cancer* **73**: 1545–1551

Remvikos Y and Mosseri V, Zajdela A, Fourquet A, Durand JC, Pouillart P and Magdelenat H (1993) Prognostic value of the S-phase fraction of breast cancers treated by primary radiotherapy or neoadjuvant chemotherapy. *Ann New York Acad Sci* **698**: 193–203

Scholl SM, Fourquet A, Asselain B, Pierga JY, Vicoq JR, Durand JC, Dorval T, Palangié T, Jouve M, Beuzeboc P, Garcio-Giratl E, Salmon RJ, De La Rochefordière A, Campana F and Pouillart P (1994) Neoadjuvant versus adjuvant chemotherapy in premenopausal patients with tumours considered too large for breast conserving surgery: preliminary results of a randomised trial: S6. *Eur J Cancer* **30A**: 645–652

Scholl SM, Asselain B, Beuzeboc P, Pierga JY, Dorval T, Garcia-Giralt E, Jouve M, Palangié T, Fourquet A, Durand JC and Pouillart P (1995) Neoadjuvant versus adjuvant chemotherapy in premenopausal patients with tumours considered too large for conserving surgery: an update (abstract P48). *Anti-Cancer Drugs* **6** (suppl. 2): 69

Scholl SM, Pierga JY, Asselain B, Buezeboc P, Dorval T, Garcia-Giralt E, Jouve M, Palangié T, Remvikos Y, Durand JC, Fourquet A and Pouillart P (1996) Breast tumour response to primary chemotherapy predicts local and distant control as well as survival. *Eur J Cancer* **31A**: 1969–1975

Semiglazov VF, Topuzov EE, Bavli JL, Moiseyenko VM, Ivanova OA, Seleznev IK, Orlov AA, Barash NY, Golubeva OM and Chepic, OF (1994) Primary (neoadjuvant) chemotherapy and radiotherapy compared with primary radiotherapy alone in stage IIb-IIIa breast cancer. *Ann Oncol* **5**: 591–595

Swenerton KD, Legha SS, Smith T, Hortobagyi GN, Gehan EA, Yap H, Gutterman JU and Blumenschein GR (1979) Prognostic factors in metastatic breast cancer treated with combination chemotherapy. *Cancer Res* **39**: 1552–1562

Tormey DC, Gray R, Abeloff MD, Roseman DL, Gilchrist KW, Barylak EJ, Stott P and Falkson G (1992) Adjuvant therapy with a doxorubicin regimen and long-term tamoxifen in premenopausal breast cancer patients: an Eastern Cooperative Oncology Group trial. *J Clin Oncol* **10**: 1848–1856

© Cancer Research Campaign 1997